# 

**Published:** 1903-11-28

**Authors:** 


### Publications.

FOURTH EDITION. Plates contain 93 Coloured and Stereoscopic Figures, 203 Illustrations in the Text, 17/6 net, including Stereoscope.

By P. WATSON WILLIAMS, M.D.(Lond.),

Physician in Charge of Throat Department, Bristol Royal Infirmary, &c., &c.

### DISEASES OF THE NOSE, PHARYNX, AND LARYNX.

WITH A SECTION ON THE EXAMINATION OF

### THE EAR.

**Press Notices.**—"We know of no better practical guide."—*Practitioner.* "Certain to receive the recommendation of all teachers."—*Brit. Med. Journ.* "We can strongly recommend the book."—*Lancet.* "Excellent.... The features of the work—completeness, clear description, and practical utility—are absolutely maintained in the new edition.—(Transn.)." *Internat. Centr. f. Laryngologie.*

Bristol: J. WRIGHT & CO. London: SIMPKIN & CO.

---

Crown 8vo. Illustrated. Price **2s. 6d.** net.

### ANÆSTHETICS. By J. Blumfeld,

M.D.Cantab., Senior Anæsthetist to St. George's Hospital; Anæsthetist to the Grosvenor Hospital for Women and Children, London, and to the National Dental and Metropolitan Hospitals.

*Lancet.*—"....In writing this handbook the author has been quite successful." *Australasian Medical Gazette.*—"We can strongly commend this book." *Medical Chronicle.*—"The book is one which we can heartily recommend to senior students and practitioners."

London: Baillière, Tindall & Cox, 8 Henrietta St., Covent Garden.

---

JUST ISSUED. EIGHTH EDITION. Crown 8vo. 10s. 6d.

### PHARMACY, MATERIA MEDICA AND THERAPEUTICS

Is now rewritten and printed in large type, and is made a companion to the following volume. It contains an account of the action **of every drug in the B.P.**, as well as a section upon all the newest drugs, with an Introduction to the Art of Dispensing. With woodcuts, prescriptions, &c.

Also FOURTH EDITION. 1055 pages, 8vo. 16s.

### DICTIONARY OF TREATMENT.

Containing a Revised and up-to-date Article upon every Diseased Condition, whether Medical, Surgical, Gynæcological, or Ophthalmological, as well as Articles upon Poisoning, Drowning, and the various Surgical Emergencies and Accidents, arranged for Rapid Reference.

By W. WHITLA, M.A., M.D., Professor of Materia Medica, Queen's College, Belfast; Senior Physician to the Royal Victoria Hospital, &c. The Publisher will supply both volumes, carriage paid, for 21/-

London: HENRY RENSHAW, 356 Strand.

---

### MEDICAL HISTORY FROM THE EARLIEST TIMES.

By E. T. WITHINGTON, M.A., M.B. Oxon.

Demy 8vo., over 400 pp., with two Plates, cloth gilt, **12/6 net.**

"One of the best attempts that has yet been made in the English language to present the reader with a concise epitome of the history of medicine, and as such we very cordially commend it." *Glasgow Medical Journal.*

THE SCIENTIFIC PRESS, Ltd., 28 and 29 Southampton Street, Strand, London, W.C.

---

WORKS BY Mr. McADAM ECCLES, M.S., F.R.C.S.,

Assistant Surgeon St. Bartholomew's Hospital, &c.

### HERNIA.

Second Edition, 1902. Price **7/6** net. Illustrated by Numerous Photographs.

### THE IMPERFECTLY DESCENDED TESTIS.

Just published. Profusely Illustrated. Price **7/6.**

London: BAILLIÈRE, TINDALL & COX.

---

JUST OUT. With 3 coloured Plates, and 54 other Illustrations. Price **6/-** net.

### HANDBOOK OF DISEASES OF THE EAR

For Students and Practitioners.

By RICHARD LAKE, F.R.C.S. Eng.,

Surgeon to the Royal Ear Hospital.

London: BAILLIÈRE, TINDALL & COX, 8 Henrietta Street, Covent Garden.

---

### CLINICAL LECTURES ON NEURASTHENIA.

By THOMAS D. SAVILL, M.D.Lond. Second Edition.

"Deals very exhaustively with the subject, and will be read with great interest."—*The Lancet.*

"It should be in the hands of every practitioner who wishes to keep himself well informed on this important subject."—*Treatment.*

H. J. GLAISHER, 57 Wigmore Street, W. 5/- net: 5/4 by post.

---

### THE HANDBOOK OF THE OPEN-AIR TREATMENT

By Dr. Charles Reinhardt

(Visiting Physician to Hailey Sanatorium, Wallingford, and Stourfield Park Sanatorium, Bournemouth),

Contains Views, Descriptions, and Information respecting 30 Sanatoria in Great Britain.

Price: Cloth, 2/6; Paper, 1/- (postage 4d.)

The "HEALTH RESORT" Publishing Office, 379 Strand, London, W.C.

---

### CLINICAL DIAGNOSIS:

**A Practical Handbook of Chemical and Microscopical Methods.**

By W. G. AITCHISON ROBERTSON, M.D., F.R.C.P. (Edin.)

Fcap. 8vo., profusely Illustrated, cloth gilt, **3/6** net, **3/9** post free.

"A handbook which accurately and succinctly offers to one who is about to undertake a chemical or microscopical investigation just those points which he ought to know, and describes exactly the methods he ought to pursue."—*The Hospital.*

THE SCIENTIFIC PRESS, Ltd., 28 & 29 Southampton Street, Strand, London, W.C.

### NOTICE TO CORRESPONDENTS.

All MSS., Letters, Books for Review, and other matters intended for the Editor should be addressed THE EDITOR, "THE HOSPITAL," THE HOSPITAL BUILDINGS, 28 & 29 SOUTHAMPTON STREET, STRAND, W.C.

The Editor cannot undertake to return rejected MS., even when accompanied by stamped directed envelope.

**Notice to Advertisers and Subscribers.**

All Advertisements, Orders for Copies of THE HOSPITAL, and Business Communications should be addressed to **THE MANAGER (Not to the Editor),** THE HOSPITAL, 28 & 29 Southampton Street, Strand, London, W.C.

The Cost of Subscription, post free, is as follows:—Per week, 3½d.; per quarter, 3s. 9d.; per half-year, 7s. 6d.; per annum, 15s. Foreign and Colonial:—Per annum, 19s. 6d., payable, in all cases, in advance.

**NOTICE.—Vol. XXXIV. of "The Hospital" (April 1903 to September 1903), in handsome cloth binding, is now on sale at the office of this Journal, price 7s. 6d. Cases for binding the Numbers contained in this Volume 1s. 6d. Index to Vol. XXXIV., 2½d., post free.**

**The Monthly part for December will be ready on December 1. Price 1s., by post, 1s. 3d.**

### BOOKS RECEIVED.

Messrs. Kegan Paul, Trench, Trübner and Co.
"A Scheme of Brain Storage." By F. R. Farmer.

Adams Brothers.
"The Antrum." Part I. By Lloyd Lavan.

Sherratt and Hughes.
"Purin Bodies of Food Stuffs." By J. Walker Hall, M.D.

American Medical Association.
"Tuberculosis of the Female Genitalia and Peritoneum." By John B. Murphy, A.M., M.D.

His Majesty's Stationery Office.
"Local Government Board Annual Report, 1902–1903."

### THE STUDENT OF MEDICINE.

### APPOINTMENTS.

**Peter H. Abercrombie**, M.D., Surgeon to the Central London Throat and Ear Hospital. **Wm. Anderson**, M.B., ChB.Aberd., Senior House Surgeon, Moorfield Eye Hospital, City Road, E.C. **G. L. Bates**, L.R.C.P., M.R.C.S., L.D.S., Dental Surgeon to the Hospital for Consumption and Diseases of the Chest, Brompton. **A. C. Black**, M.R C.S., L.R.C.P.Lond., District Medical Officer of the Malling Union. **William P. Cowper**, L.R.C.P., L.R.C.S.Edin., L.F.P.S. Glasg., Assistant Resident Medical Officer to the St. Mary's Hospital for Sick Children, Plaistow. **D. R. Powell Evans**, L.R.C.P., M.R.C.S., L.S.A., Clinical Assistant to the London Throat Hospital, Great Portland Street, W. **Thos. E. Frazer-Todvey**, F.R.C.S.Edin., Resident Medical Officer to the St. Mary's Hospital for Sick Children, Plaistow. **T. Arthur Helme**, M.D., M.R.C.P., F.R.S.E., Honorary Surgeon for Women to the Northern Hospital for Women and Children, Manchester. **Ethel Frances Lamport**, M.D.Brux., L.S.A., Public Vaccinator for the Isle of Dogs District of the Poplar Union. **R. Murray Leslie**, M.B.Edin., M.R.C.P., Physician to the Royal Hospital for Diseases of the Chest. **Jas. Webster Miller**, M.B.Aberd., Medical Officer to the Warneford Asylum, Oxford. **A. A. Myers**, M.R.C.S., L.R.C.P., Assistant Physician at the Mendip Hills Sanatorium, Wells. **W. J. Chichele Nourse**, F.R.C.S.E., Surgeon to the Central London Throat and Ear Hospital. **H. Renney**, M.D.Durh., Medical Officer of Health for Sunderland. **J. B. Ridley**, M.D.Edin., Clinical Assistant, St. John's Hospital for Skin Diseases, London, and Medical Officer, St. Olave's (London) Union Schools, Shirley, near Croydon. **David Ap. Simon**, M.B., Ch.B. Glasg., Certifying Factory Surgeon for the Nantgaredig District, Carmarthenshire. **A. Bankier Sloan**, M.D.Glasg., Honorary Extra Physician to the Dispensary, Royal Hospital for Sick Children, Glasgow. **D. Smith**, M.B., C.M.Glasg., Certifying Factory Surgeon for the Creetown District, Kirkcudbright. **C. J. Stanley**, M.B., Visiting Medical Attendant at Rochester House of the Metropolitan Asylum District. **William Stuart-Low**, F.R.C.S., Assistant-Surgeon to the Central London Throat and Ear Hospital. **Eustace Talbot**, M.B., B.C.Cantab., M.R C.P.Lond., Assistant Physician to the Royal Hospital for Diseases of the Chest. **R. Russell Thomas**, M.R.C.S., L.R.C.P., Clinical Assistant in the Ophthalmic Department, Cardiff Infirmary. **Hugh Walker**, M.A., M.B., C.M.Edin., Ophthalmic Surgeon to the Victoria Infirmary and Bellahouston Dispensary, Glasgow. **D. Wardleworth**, L.R.C.P.&S.Edin., L.F.P.S.Glasg., District Medical Officer of the Bourne Union. **Andrew Wyle**, M.D., Assistant Surgeon to the Central London Throat and Ear Hospital.

NOW READY. New and Revised Edition.

### THE UNIFORM SYSTEM OF ACCOUNTS

FOR HOSPITALS AND PUBLIC INSTITUTIONS, ORPHANAGES, MISSIONARY SOCIETIES, HOMES, CO-OPERATIONS, AND ALL CLASSES OF INSTITUTIONS.

With Special Forms of Account, Complete Sets of Books, certain suggested Checks upon Expenditure, Forms of Tender, and other Aids to Economy together with an Index of Classification whereby every item of expenditure may be dealt with under identical heads by every group of institutions.

**By SIR HENRY BURDETT, K.C.B.**

Royal 8vo, cloth boards, **4/-** net.; Post free, **4/4.**

THE SCIENTIFIC PRESS, LIMITED, 28 & 29 Southampton Street, Strand, London, W.C.

**THE PRIVATE NURSE'S OWN NOTE-BOOK.**
By SISTER EVA (with photo of Authoress). Price 6d. post free from BEMROSE & SONS, Derby.

An exceedingly well got up and useful little volume intended to fill a long felt want, containing tear-off pages for expenses incurred at cases, receipts for same, notes and valuable hints on private nursing, dainty dishes for the sick. Also spare pages for Nurse's notes.

THE SCIENTIFIC PRESS, LIMITED, 28 & 29 Southampton Street, Strand, London, W.C.

### Pocket Manuals for Nurses.

### THE EXAMINATION OF THE URINE.

By J. K. WATSON, M.D. Edin., M.B., C.M.,
Author of a "Handbook for Nurses." Bound in cloth boards, 1/- net; post free, 1/1.

### PRACTICAL GUIDE TO SURGICAL BANDAGING AND DRESSINGS.

By WM. JOHNSON SMITH, F.R.C.S.
Profusely illustrated, cloth, 2/- post free.

### NURSING NOTES ON MIDWIFERY AND GYNÆCOLOGY.

By SISTER ROSS, Rotunda Hospital, Dublin.
Cloth boards, 2/- net; 2/1 post free.

### THE NURSE'S DICTIONARY OF MEDICAL TERMS & NURSING TREATMENT.

Compiled for the use of Nurses. By HONNOR MORTEN.
Fourth and Revised Edition, bound in c'oth boards, 2/-; in handsome leather, gilt, 2/6 net; 2/8 post free.

### THE MIDWIVES' POCKET BOOK

(With Prefatory Chapter on the Midwives Bill).
By Miss HONNOR MORTEN.
Author of "The Nurse's Dictionary," &c. &c. Cloth boards, 1/- post free.

### THE POCKET CASE-BOOK FOR DISTRICT AND PRIVATE NURSES.

By a PHYSICIAN.
Cloth boards, 1/- post free; in handsome leather, gilt, 1/6 net; 1/7 post free.

*Obtainable of all Booksellers.*

THE SCIENTIFIC PRESS, LIMITED, 28 & 29 Southampton Street, Strand, London, W.C.

### WORKS FOR NURSES.

**LESSONS ON MASSAGE.** By Mrs. MARGARET D. PALMER, Manager of the Massage Department and Instructor of Massage to the Nursing Staff of the London Hospital. Second Edition enlarged and revised; pp. xvi. + 262 with 118 Illustrations, plain and coloured. *Just Published.* Price 7/6 net.

**A MANUAL FOR STUDENTS OF MASSAGE.** By Miss M. A. ELLISON, L.O.S. With 50 original Illustrations. Price 3/6 net.

**A MANUAL FOR HOME NURSING.** By BERNARD MYERS, M.D., M.R.C.S., Surgeon and Lecturer to St. John's Ambulance Association. *Just Published.* Price 2/6 net.

**NOTES ON HOME NURSING.** By Miss D. M. GOLDIE, Lecturer on Nursing, Hygiene, &c., to the County Councils of London, Surrey and Middlesex. *Just Published.* Price 1/6 net.

**THE FEEDING AND HYGIENE OF INFANTS AND CHILDREN.** By JOHN McCAW, M.D., M.R.C.P., Physician to the Belfast Hospital for Children. *Just Published.* Price 2/6.

**DISEASES OF THE HAIR, INCLUDING GREYNESS, BALDNESS, &c., AND THEIR TREATMENT.** By DAVID WALSH, M.D., Western Hospital for Diseases of the Skin. Price 2/6 net.

**MENSTRUATION AND ITS DISORDERS.** By ARTHUR E. GILES M.D., B.Sc., F.R.C.S., Surgeon Chelsea Hospital for Women. Price 2/6 net.

**MOVABLE ATLAS OF THE ANATOMY AND PHYSIOLOGY OF THE FEMALE GENERATIVE ORGANS AND OF PREGNANCY.** Second Edition. With Text. By ARTHUR E. GILES, M.D., F.R.C.S. Price 3/- net.

**MOVABLE ATLAS OF THE ANATOMY AND PHYSIOLOGY OF THE CHILD.** With Explanatory Text. By D'ARCY POWER, M.A.Oxon, M.B., F.R.C.S. Price 3/- net.

**BAILLIÈRE'S POPULAR MOVABLE ATLAS OF THE ANATOMY AND PHYSIOLOGY OF MAN.** Size 16 by 7½ inches. With Explanatory Text. By W. S. FURNEAUX, Special Science Teacher London School Board. Price 3/6 net.

London: BAILLIÈRE, TINDALL & COX, 8 Henrietta Street, Covent Garden.

### NOW READY,

A REPRINT OF

### A NURSING HANDBOOK

TOGETHER WITH S. MAW, SON AND SONS'

### COMPLETE CATALOGUE OF NURSING REQUISITES.

The above work is now republished at a cost of 1s. per copy. Messrs. S. M., S. and S. have been compelled to make this charge on account of the extreme popularity of the work and the great expense incurred in its production.

***Post Free on receipt of 1s.***

Nurses' Bags.

### S. MAW, SON and SONS,

**7 to 12 ALDERSGATE STREET, LONDON, E.C.**

Standard Works

### J. & A. CHURCHILL'S

### Books for Nurses.

Latest Editions

**NURSING.**

**Nursing, General, Medical and Surgical, with an Appendix on Sick Room Cookery.** By WILFRED J. HADLEY, M.D., F.R.C.P., F.R.C.S., Physician and Pathologist to the London Hospital, Lecturer to Nurses at the London Hospital Nursing School . . . . 3s. 6d.

**MEDICINE.**

**Lectures on Medicine to Nurses.** By HERBERT E. CUFF, M.D., F.R.C.S., Medical Superintendent, North Eastern Fever Hospital, London. Fourth Edition. With 29 Illustrations . . . . . . 3s. 6d.

**INFANT FEEDING.**

**The Natural and Artificial Methods of Feeding Infants and Young Children.** By EDMUND CAUTLEY, M.D., F.R.C.P., Physician to the Belgrave Hospital for Children; late Casualty Physician and Assistant Demonstrator of Physiology, St. Bartholomew's Hospital. Second Edition. 7s. 6d.

**DIET.**

**Diet and Food considered in relation to Strength and Power of Endurance, Training and Athletics.** By A. HAIG, M.D., F.R.C.P. Fourth Edition. 2s.

**MIDWIFERY.**

**A Short Practice of Midwifery for Nurses.** By HENRY JELLETT, M.D. (Dub. Univ.), F.R.C.P.I., Ex-Assistant Master, Rotunda Hospital, Dublin, Extra Examiner in Midwifery and Gynæcology, Royal College of Physicians of Ireland, Extern Examiner in Midwifery, Royal University of Ireland. With 67 Illustrations . . . . 6s.

**MONTHLY NURSING.**

**A Short Manual for Monthly Nurses.** By C. J. CULLINGWORTH, M.D., F.R.C.P., Obstetric Physician to St. Thomas's Hospital. Revised with the assistance of M. A. ATKINSON, Matron of the General Lying-in Hospital, London. Fifth Edition. 1s. 6d.

**HEALTH OF A WIFE.**

**Chavasse's Advice to a Wife on the Management of her Own Health.** Fourteenth Edition. By FANCOURT BARNES, M.D., Consulting Physician to the British Lying-in Hospital . . . . . 2s. 6d.

**HEALTH OF CHILDREN.**

**Chavasse's Advice to a Mother on the Management of her Children.** Fifteenth Edition. By GEORGE CARPENTER, M.D., Physician to the Evelina Hospital for Children . . . . . . 2s. 6d.

**ANTISEPTICS.**

**Lessons in Disinfection and Sterilisation; an Elementary Course of Bacteriology, with a Scheme of Practical Experiments.** By F. W. ANDREWES, M.D., F.R.C.P., Lecturer on Pathology at St. Bartholomew's Hospital. With Illustrations . 3s. net.
[*Just Published.*

**SURGERY.**

**A Manual of Minor Surgery and Bandaging.** By CHRISTOPHER HEATH, F.R.C.S., and BILTON POLLARD, F.R.C.S., Surgeon to University College Hospital. Twelfth Edition. With 195 Illustrations. 6s. 6d.

**HYGIENE.**

**The Elements of Health: An Introduction to the Study of Hygiene.** By LOUIS C. PARKES, M.D., D.P.H.Lond., Lecturer on Public Health at St. George's Hospital. 3s. 6d.

**DICTIONARY.**

**A Short Dictionary of Medical Terms.** By R. G. MAYNE M.D., LL.D. . 2s. 6d.

**7 GREAT MARLBOROUGH STREET, LONDON.**

